# Propofol reduces synaptic strength by inhibiting sodium and calcium channels at nerve terminals

**DOI:** 10.1007/s13238-019-0624-1

**Published:** 2019-04-26

**Authors:** Qing-Zhuo Liu, Mei Hao, Zi-Yang Zhou, Jian-Long Ge, Yi-Chen Wu, Ling-Ling Zhao, Xiang Wu, Yi Feng, Hong Gao, Shun Li, Lei Xue

**Affiliations:** 1 State Key Laboratory of Medical Neurobiology, Department of Physiology and Biophysics, School of Life Sciences and Collaborative Innovation Centre for Brain Science, Fudan University, Shanghai, 200438 China; 2 The Affiliated Hospital of Medical College, Ningbo University, Ningbo, 315020 China; 3 Department of Critical Care Medicine, Shanghai General Hospital, Shanghai Jiaotong University, Shanghai, 200080 China; 4 Department of Orthopaedic Surgery, Shanghai Jiaotong University Affiliated Sixth People’s Hospital, Shanghai, 200233 China; 5 Zhejiang Provincial People’s Hospital, Hangzhou, 310000 China

**Dear Editor,**

Propofol (2,6-diisopropylphenol) has been widely used in clinical surgery because of its fast induction and rapid recovery effects (Miller et al., 2014). Although the underlying mechanisms are still controversial, many studies have focused on the augmentation of GABA-induced inhibition and/or modulation of glycine receptor channel activity. However, inhibition of glutamatergic synaptic transmission may also contribute to general anesthesia. The effects of general anesthetics are significantly enhanced when glutamate receptor antagonists are co-administered (Ishizaki et al., 1996). Intrathecal administration of both GABA_A_ and glycine receptor antagonist only increase the minimum alveolar concentration ~50%, suggesting that enhanced inhibitory synaptic transmission is not sufficient to account for general anesthesia (Zhang et al., 2001). However, the underlying mechanisms by which intravenous anesthetics inhibit glutamatergic synaptic transmission remain unclear.

Exocytosis and endocytosis play crucial physiological roles in neuronal communication. Inhibition of exocytosis directly inhibits the postsynaptic current, and inhibition of endocytosis exhausts the secretion-competent vesicles, both of which suppress synaptic transmission. General anesthetics may play a role in the inhibition of each of these steps, but precise presynaptic mechanisms are lacking. A previous study showed that isoflurane inhibits the excitatory postsynaptic current (EPSC) by reducing presynaptic action potential amplitude (Wu et al., 2004). Whether general anesthetics inhibit presynaptic calcium and subsequent exo-endocytosis remains elusive. The contribution of each presynaptic step needs to be clarified further. Here, we studied how propofol modulates synaptic transmission at a giant glutamatergic synapse, the calyx of Held, in the rat brainstem. The large presynaptic nerve terminal allows for direct measurement of the presynaptic action potential, calcium influx, and vesicle exo-endocytosis and the combination of both pre- and post-synaptic recordings may provide new insights into the propofol-modulated synaptic transmission.

First, we evoked postsynaptic EPSCs by fiber stimulation at the midline of the trapezoid body every 10 s. After obtaining a stable baseline for 5 min, we added 250 μmol/L propofol to the extracellular solution. The EPSCs were significantly reduced by 14% ± 1% in approximately 5 min (*n* = 7; Fig. 1A and 1B), showing an acute inhibitory effect by propofol. We also examined the inhibition of the EPSC with different propofol concentrations from 100–500 μmol/L. We did not observe significant inhibition at 100 μmol/L propofol (*n* = 5; Fig. 1A and 1B). However, at concentrations of 500 μmol/L, we observed increased inhibition of the EPSC amplitude (*n* = 5; Fig. 1A and 1B). At all concentrations of propofol, the rise time and decay time of the EPSCs were not affected (Fig. 1B).

**Figure 1:**
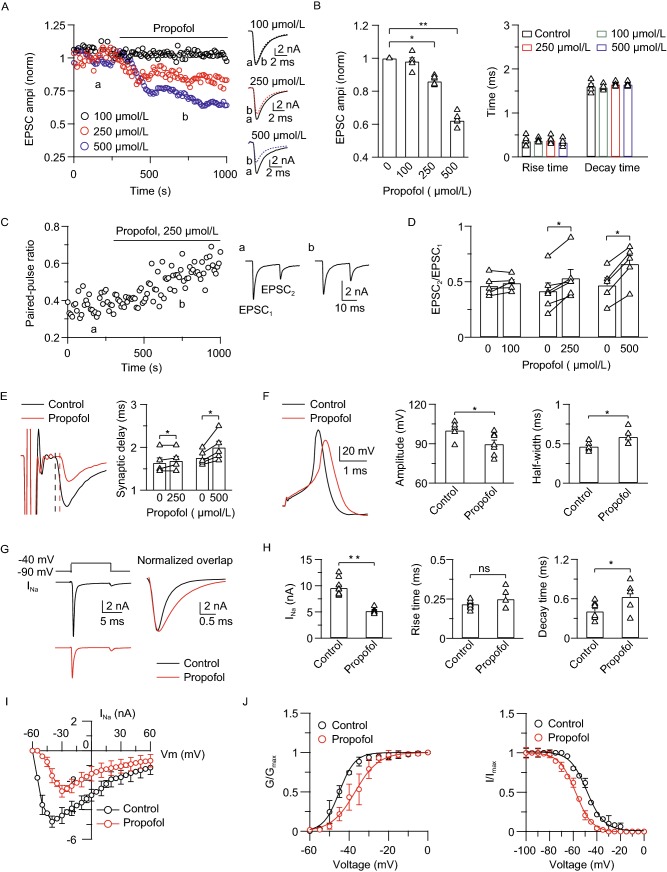
**Propofol reduces EPSC amplitude by inhibiting the presynaptic sodium current**. (A) Left: EPSC was recorded every 10 s after stimulation at the midline of the trapezoid body. 100–500 μmol/L propofol was added to the bath solution after obtaining a stable baseline and recorded for another ~15 min (100 μmol/L, black; 250 μmol/L, red; 500 μmol/L, blue). Right: Sampled EPSCs from time points a and b were superimposed. (B) Left: The mean normalized EPSC amplitude after administration of propofol at different concentrations (100–500 μmol/L). The EPSC was averaged from 30 EPSCs 5 min after application of propofol and normalized to the current averaged from 30 EPSCs before the application of propofol. Right: The mean 10%–90% rise time (control, *n* = 5; propofol, *n* = 5–7) and decay time (control, *n* = 5; propofol, *n* = 5–7 for each group) before and after administration of propofol. *P < 0.05; **P < 0.01. (C) Left: The paired-pulse ratio (PPR, EPSC_2_/EPSC_1_) was plotted before and after administration of 250 μmol/L propofol. Right: Sampled pair of EPSCs from time points a and b. (D) The mean PPR before and after administration of 100, 250 and 500 μmol/L propofol. *P < 0.05. (E) Left: Sampled EPSCs from control (black) and propofol-treated (red) calyces. Traces are aligned to show the synaptic delay, and the dashed line indicates the onset of the EPSC. Right: Statistics for the synaptic delay from the control and propofol-treated groups (250 and 500 μmol/L). *P < 0.05. (F) Left: Sampled action potential waveform induced by single current injection (1-ms step current of 500 pA). Black, control; red, propofol-treated. Middle and right: Statistics for the action potential amplitude and half-width in controls and propofol-treated calyces. *P < 0.05. (G) Left: Sampled sodium current induced by a 10-ms depolarization pulse from −90 to −40 mV. Black, control; red, propofol-treated. Right: Overlap of the sampled sodium current traces from the control (black) and propofol-treated (scaled, red) groups. (H) Statistics for sodium current amplitude, 10%–90% rise time, and decay time in control and propofol-treated calyces. *P < 0.05; **P < 0.01. (I) Plot of the I-V sodium current induced by a 10-ms depolarization from −60 to +60 mV with a step of 5 mV in control (black) and propofol-treated calyces (red, *n* = 4–5 for each data point). (J) Left: Sodium current activation curves. Right: Sodium current inactivation curves. Black, controls; red, propofol-treated calyces (*n* = 4–5 for each data point)

Next, we measured the paired-pulse ratio (PPR) to further examine whether inhibition of the EPSC was caused by a presynaptic mechanism. A pair of stimuli with an interval of 20 ms induced two consecutive EPSCs at the principal neuron. After administration of 250 μmol/L propofol, the PPR significantly increased to 53% ± 8% (*n* = 6; Fig. 1C). With 100 μmol/L propofol, we did not observe a significant change in the PPR (*n* = 5; Fig. 1D). However, with 500 μmol/L propofol, the PPR was further increased to 66% ± 8% (*n* = 5; Fig. 1D). In addition to the increased PPR, we also observed an increase in synaptic delay after the administration of propofol (Fig. 1E), suggesting a slowdown in the synaptic transmission arriving at the principal neuron. We further recorded the mEPSCs and found propofol does not affect the sensitivity of postsynaptic AMPA receptors (Fig. S1). The increased PPR and prolonged synaptic delay suggest that the inhibition of EPSCs is caused by presynaptic mechanisms.

The release of presynaptic glutamate is initiated by the arrival of action potentials at the nerve terminal. By whole cell current injection, we found that the AP amplitude was decreased by ~10% (control: 100.2 ± 2.8 mV, *n* = 5; propofol: 89.7 ± 2.9 mV, *n* = 7) and the half-width increased by ~26% (control: 0.47 ± 0.02 ms, *n* = 5; propofol: 0.59 ± 0.04 ms, *n* = 5; Fig. 1F) with 250 μmol/L propofol, suggesting a direct modulation of the AP waveform. Because the AP amplitude is determined mostly by the sodium channel, we measured the sodium currents by blocking the calcium and potassium channel-mediated currents. A 10-ms depolarization pulse from −90 to −40 mV induced a large sodium current (I_Na_) of 9.6 ± 0.5 nA (*n* = 9). With 250 μmol/L propofol, the I_Na_ was reduced to 5.2 ± 0.3 nA (*n* = 5; Fig. 1G and 1H), which is in agreement with a previous study in the hippocampal synaptosome (Lingamaneni et al., 2001). The rise time of I_Na_ was not affected (*n* = 5). However, the decay time was increased after administration of 250 μmol/L propofol (control: 0.41 ± 0.04 ms, *n* = 9; propofol: 0.63 ± 0.10 ms, *n* = 5; Fig. 1H). The current-voltage (I-V) curve showed that the sodium current was reduced at every voltage step, and the peak shifted to the right (Fig. 1I). The sodium current activation/inactivation curves also demonstrated significant modulation of sodium channel (Fig. 1J), which is consistent with the increased synaptic delay. These results suggest that the reduced I_Na_ amplitude inhibited the AP amplitude, whereas increased decay time increased the half-width.

Propofol inhibited the EPSC largely due to the reduction in calcium influx (see Fig. S3 for the quantitative relationship). We plotted the I-V curve induced by a 200-ms depolarization pulse from −80 mV to +40 mV with an interval of 30 s (Fig. 2A). With 250 μmol/L propofol, the calcium current was smaller than in controls at every voltage step, and the peak did not shift (Fig. 2A), suggesting that propofol does not affect the characteristics of calcium channels. In p8–p10 rats, P/Q-, N- and R-type calcium channels are expressed at the presynaptic nerve terminal (Iwasaki et al., 2000). To determine which subtype accounted for inhibition of the calcium current, we applied calcium channel blockers to examine whether the remaining calcium current was vulnerable to propofol. When 200 nmol/L omega-agatoxin IVA, the P/Q-type calcium channel blocker, was applied to the bath solution for 30 min, the calcium current was reduced to 0.8 ± 0.1 nA, ~38% of control (*n* = 5). We then added 250 μmol/L propofol for another 30 min and found the calcium current was not reduced further (0.9 ± 0.1 nA, *n* = 5; Fig. 2B). When 1 µmol/L omega-conotoxin and 100 nmol/L SNX-482, the N- and R-type calcium channel blockers, were applied to the bath solution for 30 min, the calcium current was reduced to 1.4 ± 0.1 nA, ~70% of control (*n* = 5). Application of 250 μmol/L propofol for another 30 min further reduced the calcium current to 0.8 ± 0.1 nA (*n* = 5; Fig. 2C), showing a partial block of the P/Q-type calcium channel current. These results suggest that the P/Q-type calcium channel may be the main target mediating the propofol-inhibited calcium current at p8o–p10 calyces.

**Figure 2:**
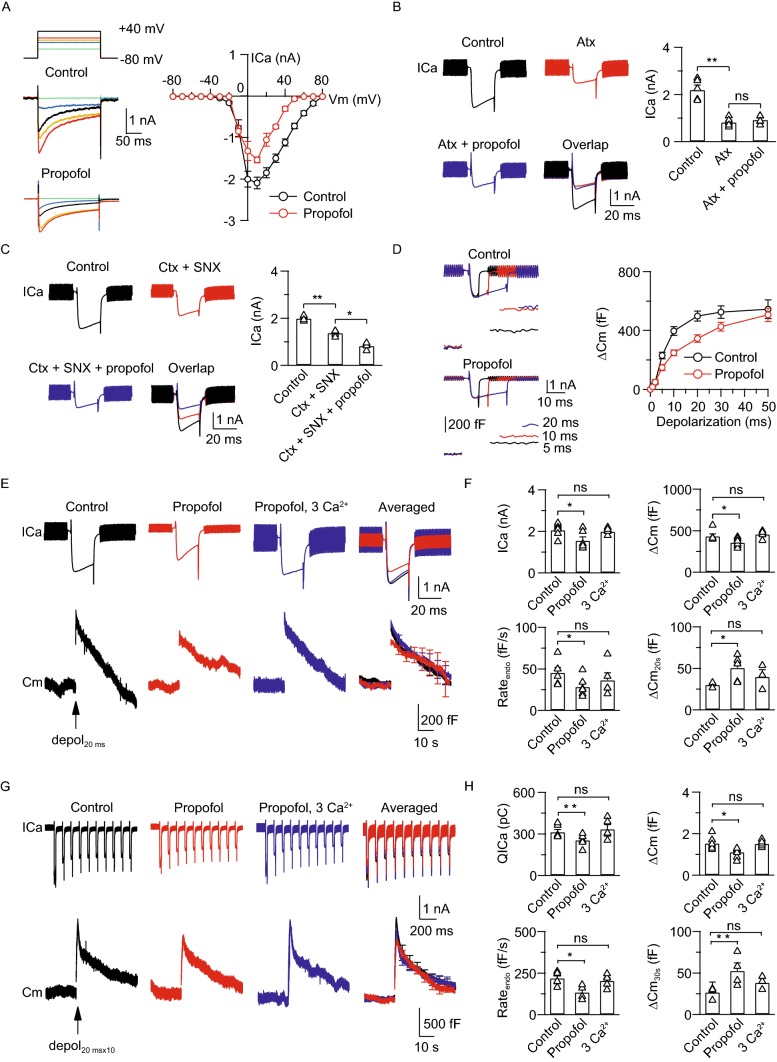
**Propofol inhibits presynaptic calcium current, exocytosis and endocytosis**. (A) Left: Sampled calcium currents in response to 200 ms depolarization from −80 to +40 mV with a step of 10 mV in control and propofol-treated calyces. Right: Plot of the I-V relationship in control (black) and propofol-treated calyces (red, *n* = 5–9 for each data point). (B) Left: Sampled calcium current in controls (black), in the presence of omega-agatoxin-IVA (200 nmol/L, red), or in the presence of propofol (250 μmol/L) and omega-agatoxin-IVA (blue) calyces and the sampled traces are superimposed. Right: Statistics for ICa in left. **P < 0.01. (C) Left: Sampled calcium current in controls (black), in the presence of omega-conotoxin (1 µmol/L) and SNX-482 (100 nmol/L, red), or in the presence of propofol (250 μmol/L), omega-conotoxin, and SNX-482 (blue) calyces and the sampled traces are superimposed. Right: Statistics for ICa in left. *P < 0.05; **P < 0.01. (D) Left: Sampled ICa and Cm induced by depolarization steps from −80 to +10 mV of various lengths (5, 10 and 20 ms) in the control and propofol group. The time scale applied to all traces. Right: Relationship between capacitance jump and the duration of step depolarization (control, black; propofol, red). (E) Left: Sampled presynaptic calcium current (ICa, upper) and membrane capacitance (Cm, lower) induced by depol_20ms_ in controls. Middle left: Similar to left, but for the propofol-treated calyces. Middle right: Similar to middle left, but with 3 mmol/L extracellular calcium. Right: Averaged ICa and Cm are superimposed to show the difference (control: *n* = 6; propofol: *n* = 6; propofol with high calcium: *n* = 5). (F) Statistics for ICa, ΔCm, Rate_endo_, and ΔCm_20s_% in controls, propofol-treated calyces, and propofol-treated calyces with 3 mmol/L extracellular calcium. *P < 0.05. (G) Left: Sampled ICa (upper) and Cm (lower) induced by depol_20msx10_ in controls. Middle left: Similar to left, but for the propofol-treated calyces. Middle right: Similar to middle left, but with 3 mmol/L extracellular calcium. Right: Averaged ICa and Cm are superimposed to show the difference (control: *n* = 6; propofol: *n* = 8; propofol with high calcium: *n* = 5). (H) Statistics for QICa, ΔCm, Rate_endo_, and ΔCm_30s_% in control and propofol-treated calyces. *P < 0.05

Since calcium/calmodulin triggers exocytosis and initiates all forms of endocytosis (Wu et al., 2009), we next measured the calcium influx and vesicle exo-endocytosis. By applying stimulation pulses of various lengths (1 to 50 ms) to induce exocytosis and measure the readily releasable pool (RRP) size, we found that propofol decreases the vesicle release probability without affecting the RRP size (Fig. 2D). We have previously shown that depol_20ms_ and depol_20msx10_ can induce clathrin-dependent and -independent endocytosis, respectively (Sun et al., 2016). With 250 μmol/L propofol, depol_20ms_ induced a mean calcium influx and exocytosis of 1.6 ± 0.2 nA and 359 ± 21 fF (*n* = 6), which was significantly smaller than in controls (calcium influx: 2.1 ± 0.1 nA; exocytosis: 435 ± 27 fF; *n* = 6; Fig. 2E and 2F). The subsequent endocytosis was also slowed. The Rate_endo_ after depol_20ms_ was reduced (control: 46 ± 6 fF/s, *n* = 6; propofol: 29 ± 5 fF/s, *n* = 6; Fig. 2E and 2F). The residual capacitance measured 20 s after depol_20ms_ was higher than in controls (Fig. 2F). Similarly, with 250 μmol/L propofol, the total calcium influx induced by depol_20msx10_ was also significantly reduced with a QICa of 256 ± 18 pC (*n* = 6), accompanied by a reduction in vesicle exocytosis (1,112 ± 81 fF, *n* = 8; Fig. 2G). The Rate_endo_ was dramatically inhibited and the residual capacitance measured 30 s after depol_20msx10_ was much higher (Fig. 2H). Thus, we found that propofol can impair presynaptic vesicle exocytosis and both rapid and slow endocytosis. Furthermore, when the extracellular calcium concentration was increased to 3 mmol/L, the propofol-inhibited calcium influx, exo- and endo-cytosis, and the postsynaptic EPSC could be fully counterbalanced, indicating a predominate calcium-dependent presynaptic mechanism (Figs. 2E–H and S4).

The clinical dose of propofol on human is up to ~30–100 μmol/L (Haeseler et al., 2001; Iida et al., 2006), lower than 250 μmol/L used in the present study. Several lines of evidence may help explain the discrepancy. First, the unconsciousness dose in rats has been reported to be ~3 times higher than human (Cotten et al., 2009). Second, our experiments were performed at 22–24 °C, whereas the temperature in clinical use is close to body temperature. The blood concentration of propofol is ~28% higher during hypothermia than in normothermia (Bissonnette, 2011). Third, in the brain slice recordings, the exact concentration that reached the cell surface would be lower because of the neural fibers inside the slice. A previous study reported an ~3.4 times lower estimated with KCl (He et al., 2010).

Calcium-influx induced synaptic exocytosis initiates synaptic transmission. Whether machinery downstream of calcium could also be potential targets from general anesthetics remains unclear. A recent study reported that propofol impairs neurotransmitter release by restricting the mobility of syntaxin 1A in cultured neurons (Bademosi et al., 2018). Since the syntaxin 1A-mediated neurotransmission is highly conserved from worms to humans, it would be essential to see whether propofol could also affect other soluble NSF-attachment protein receptor (SNARE) proteins and how they inhibit vesicle fusion in the future studies.

In summary, we examined the presynaptic mechanisms of propofol-inhibited glutamatergic synaptic transmission at a central synapse. Propofol can inhibit the presynaptic sodium and calcium channels, both of which suppress the calcium current, resulting in substantial reduction of exocytosis and the EPSC, with a slowing down of endocytosis. Our study may provide helpful information on the clinical use of general anesthetics.

## Electronic supplementary material

Below is the link to the electronic supplementary material.

Supplementary material 1 (PDF 580 kb)
