# Supplementary material for: Propofol reduces synaptic strength by inhibiting sodium and calcium channels at nerve terminals
Source: Protein Cell. 2019 Apr 26;10(9):688–93. doi: 10.1007/s13238-019-0624-1 (PMC6711943; doi:10.1007/s13238-019-0624-1)
Supplement: Supplementary file 1 — Supplementary material 1 (PDF 580 kb) [file 13238_2019_624_MOESM1_ESM.pdf]

Propofol reduces synaptic strength by inhibiting sodium and calcium channels at nerve terminals

Running title: Presynaptic mechanisms of propofol on synaptic transmission

Qing-zhuo Liu[1*], Mei Hao[1*], Zi-yang Zhou[1*], Jian-long Ge[1], Yi-chen Wu[1], Ling-ling Zhao[1], Xiang Wu[2], Yi Feng[3], Hong Gao[4], Shun Li[5]#, and Lei Xue[1]#

1. State Key Laboratory of Medical Neurobiology, Department of Physiology and Biophysics, School of Life Sciences and Collaborative Innovation Centre for Brain Science, Fudan University, Shanghai, P.R.China, 200438
2. The Affiliated Hospital of Medical College, Ningbo University, Ningbo, Zhejiang Province, P.R.China, 315020
3. Department of Critical Care Medicine, Shanghai General Hospital, Shanghai Jiaotong University, Shanghai, P.R.China, 200080
4. Department of Orthopaedic Surgery, Shanghai Jiaotong University Affiliated Sixth People's Hospital, Shanghai, P.R.China, 200233
5. Zhejiang Provincial People's Hospital, Hangzhou, Zhejiang Province, P.R.China, 310000

Corresponding author: Dr. Lei Xue or Dr. Shun Li

Email: lxue@fudan.edu.cn or 1148449287@qq.com

Supplementary Information

I. Materials and Methods

Slice preparation

Sprague-Dawley rats (8 - 10 days old, p8 - p10) of either sex were decapitated and tissue blocks containing the MNTB placed in a low-Ca^{2+} artificial cerebrospinal fluid (ACSF) solution (125 mM NaCl, 25 mM $NaHCO_3$, 3 mM myo-inositol, 2 mM Na-pyruvate, 2.5 mM KCl, 1.25 mM NaH_2PO_4, 0.4 mM ascorbic acid, 25 mM glucose, 3 mM $MgCl_2$, and 0.05 mM $CaCl_2$). Brain slices (~200 μm thick) were sectioned using a vibratome (VT 1200s, Leica, Germany) and recovered in normal ACSF with 2 mM $CaCl_2$ at 37 ℃ for 30 - 40 min before experiments. Propofol was diluted in dimethylsulfoxide (DMSO) at a concentration of 50

mM and stored frozen (-20 ℃). For use, the stock solution was vortexed to ensure complete mixing of components and diluted into the extracellular solution to a final working concentration of 100 - 500 μM. The DMSO concentration of 0.1% was maintained in all solutions. All electrophysiological recordings were made at room temperature (22 - 24 ℃).

All of the methods were carried out in accordance with the approved guidelines, and all animal experimental protocols were approved by the Animal Care and Use Committee of Fudan University.

Electrophysiology

The postsynaptic EPSC and miniature EPSC (mEPSC) were recorded by an EPC-10 amplifier (HEKA, Lambrecht, Germany). The pipette (2 - 3 MΩ) solution contained 125 mM K-gluconate, 20 mM KCl, 4 mM Mg-ATP, 10 mM Na_2-phosphocreatine, 0.3 mM GTP, 10 mM HEPES, and 0.5 mM EGTA (pH 7.2, adjusted with KOH). The series resistance (< 10 MΩ) was compensated by 95% (lag 10 μs). mEPSCs were recorded at a holding membrane potential of -80 mV with 0.5 μM tetrodotoxin (TTX). Bicuculline (10 μM) was added to the bath solution to inhibit $GABA_A$-mediated current. The EPSC was induced by a 0.1 ms, 2-20 V pulse applied via a bipolar electrode placed at the midline of the trapezoid body in the presence of 100 μM cyclothiazide (CTZ) to avoid desensitization of AMPA receptors.

The presynaptic membrane capacitance measurements were made using an EPC-10 amplifier with lock-in software. The presynaptic pipette (3.5 - 5 MΩ) solution contained 125 mM Cs-gluconate, 20 mM CsCl, 4 mM Mg-ATP, 10 mM Na_2-phosphocreatine, 0.3 mM GTP, 10 mM HEPES, and 0.05 mM BAPTA (pH 7.2, adjusted with CsOH). The series resistance (< 10 MΩ) was compensated by 65% (lag 10 μs). When calcium channel blockers were applied to the bath, cytochrome c (0.1 mg/ml) was also included. Slow endocytosis was induced by a 20-ms depolarization pulse from -80 mV to +10 mV ($depol_{20ms}$), and rapid endocytosis was induced by 10 $depol_{20ms}$ at 10 Hz ($depol_{20msx10}$). Action potentials were

induced by single current injection (1 ms step current of 500 pA) or trains (10 pulses at 100 Hz). The isolated presynaptic sodium current was recorded using a cesium-based internal solution with 200 μM $CdCl_2$ and 20 mM TEA-Cl in the ACSF to block calcium and potassium channels. The series resistance was compensated by 95-98%. When plotting the activation/inactivation gating kinetics, 10 nM TTX was added to the bath solution to partially inhibit the sodium current. The sodium current-voltage (I-V) curves were recorded by applying 10 ms depolarizing pulses from −60 to +60 mV at 5 mV steps. The steady-state activation I_{Na} was obtained as described above. The steady-state inactivation of I_{Na} was obtained by a double-pulse protocol composed of a 100-ms conditioning pre-pulse from -100 to 0 mV with 5 mV increment and a following 12 ms pulse from -70 to -10 mV. Isolated presynaptic potassium current was recorded with 200 μM $CdCl_2$ and 0.5 μM TTX in the ACSF to block calcium and sodium channels.

Simultaneous pre- and postsynaptic recordings were performed with an EPC10 double amplifier. The mimicked action potentials were generated by Patchmaster software (HEKA, Lambrecht, Germany).

Data analysis

Synaptic delay was measured between the onset of the presynaptic stimulation artefact and the oneset of the postsynaptic EPSC. The mEPSCs were analyzed by the Mini Analysis Program (version 6.07, Synaptosoft, USA). Capacitance jumps were measured 250 ms after depolarization to avoid artifacts. The initial rate of endocytosis ($Rate_{endo}$) was measured within 2 s after depolarization with $depol_{20ms}$ or $depol_{20msx10}$. The residual capacitance 20 s after $depol_{20ms}$ (ΔCm_{20s}) or 30 s after $depol_{20msx10}$ (ΔCm_{30s}) was measured to represent endocytosis recovery. All data were expressed as mean ± SE. P-values were determined by t-test, and $p < 0.05$ was considered significant.

II. Propofol does not affect the sensitivity of postsynaptic AMPA receptors

We recorded the postsynaptic mEPSCs to investigate whether propofol can directly modulate the sensitivity of postsynaptic AMPA receptors. All mEPSCs were recorded at a holding potential of -80 mV with 250 μM propofol in the extracellular solution. We also added 10 μM bicuculline to an individual group to block the modulation of $GABA_A$ receptor for comparison. The mean mEPSC amplitude and frequency in controls was 28.2 ± 1.4 pA and 1.2 ± 0.2 Hz (920 events, n = 5; Fig. S1a-c). The mean 10-90% rise time and decay time were 0.61 ± 0.03 ms and 0.83 ± 0.08 ms, respectively (Fig. S1d). Administration of 250 μM propofol did not change the amplitude (29.8 ± 2.6 pA, 732 events, n = 5, p = 0.7) or frequency (1.0 ± 0.2 Hz, n = 5, p = 0.6; Fig. S1a-c). Combining propofol and bicuculline still did not change the mEPSC amplitude (31.0 ± 2.8 pA, 768 events, n = 5, p = 0.4) or frequency (1.1 ± 0.1 Hz, n = 5, p = 0.8; Fig. S1a-c). The mean 10-90% rise time or decay time was also not significantly different from controls in the propofol or propofol plus bicuculline group (propofol: rise time 0.62 ± 0.06 ms, p = 0.4, decay time 1.01 ± 0.13 ms, p = 0.9; propofol + bicuculline: rise time 0.67 ± 0.03 ms, p = 0.7, decay time 0.97 ± 0.05 ms, p = 0.8; Fig. S1d). The mEPSC recordings suggest that propofol does not affect the sensitivity of the postsynaptic AMPA receptors.

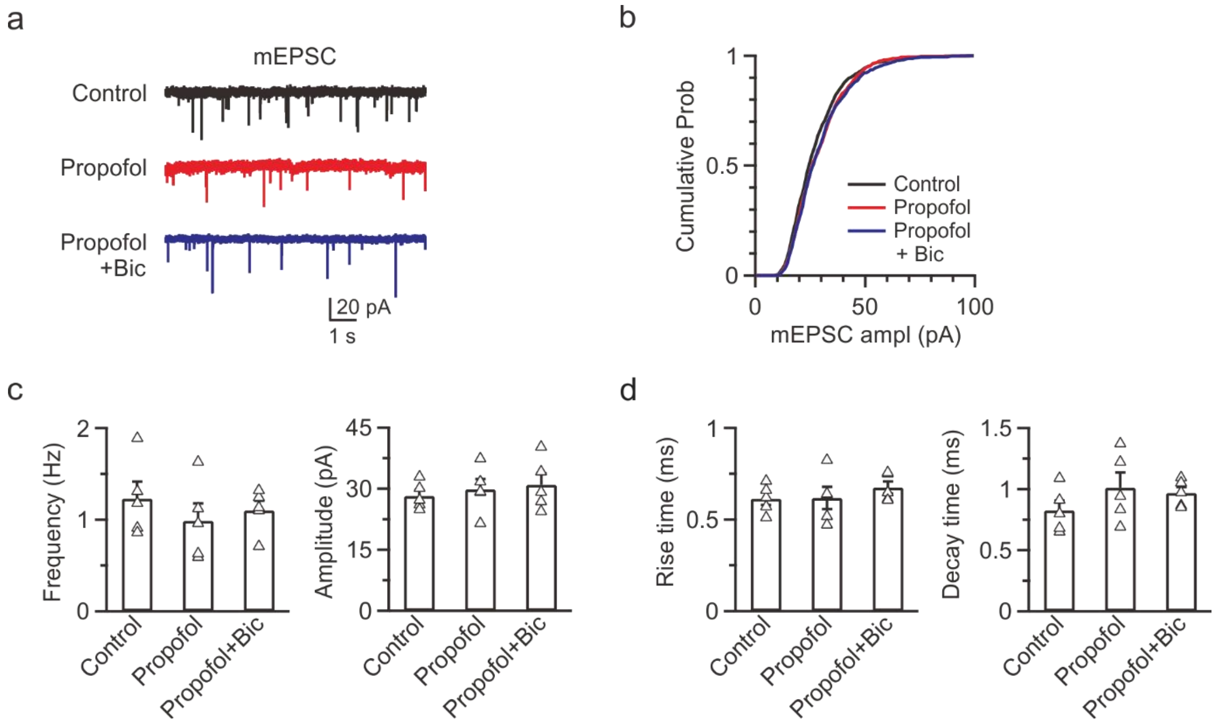

Figure S1. Propofol does not affect the sensitivity of postsynaptic AMPA receptors

a) Sampled mEPSC recordings from control (upper, black), propofol-treated (middle, red), and propofol-treated with bicuculline calyces (lower, blue).

b) The cumulative probability distribution of mEPSCs in controls (black), propofol-treated (red), and propofol-treated with bicuculline calyces (blue).

c) Statistics for the frequency and amplitude of mEPSCs in controls, propofol-treated, and propofol-treated with bicuculline calyces.

d) Statistics for the 10-90% rise time and decay time of mEPSCs in controls, propofol-treated, and propofol-treated with bicuculline calyces.

III. Propofol does not affect the potassium current

To determine whether propofol can affect potassium current, we recorded the potassium current by applying a 50 ms depolarization pulse from -80 mV to -60, -40, -20, 0, and 20 mV, respectively (Ishikawa et al., 2003; Nakamura and Takahashi, 2007). After administration of 250 μM propofol, a 50 ms depolarization pulse induced progressively increased potassium currents which were not significantly different compared to the controls (Fig. S2), suggesting a minimal effect on the potassium current.

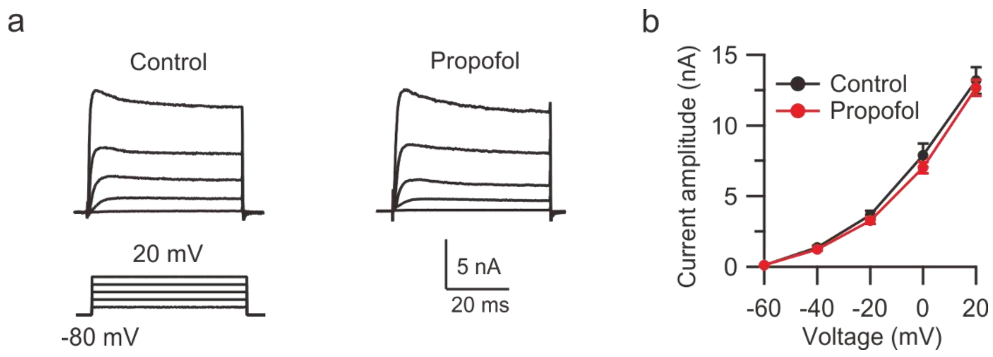

Figure S2. Propofol does not affect the potassium current

a) Left: Sampled potassium currents (upper) in controls induced by a 50 ms depolarization from -80 mV to -60, -40, -20, 0, and 20 mV (lower), respectively. Right: Similar to left, but for the propofol group.
b) The relationship between the amplitude of the potassium current and depolarization voltage (control, black; propofol, red).

IV. Relationship between the EPSC and presynaptic action potential waveform

Inhibition of the action potential amplitude has been reported to critically affect the EPSC by a power of 10.2, a highly non-linear relationship (Wu et al., 2004). Based on this model, our 10-15% reduction in AP amplitude predicts a 70-80% decrease in the postsynaptic EPSC, which is different from the 10-20% decrease we observed in experiments (see main text Fig. 1A). The discrepancy was due to the increased AP width, which would partially counterbalance the inhibitory effect on EPSC amplitude. To further clarify the relationship, we examined the postsynaptic EPSC amplitude in different AP waveform scenarios. An experimentally obtained AP of 130 mV evoked by fiber stimulation at the midline of the trapezoid body was chosen as the voltage command to mimic an AP (Xue and Wu, 2010), which would induce a presynaptic calcium current and the corresponding postsynaptic EPSC (1.2 ± 0.5 nA, n = 4; Fig. S3a, left). When we reduced the AP amplitude by 15% and left the width unchanged, the EPSC was dramatically reduced to 18 ± 5% of control (n = 4; Fig. S3a, middle left), which matches the previous model (Wu et al., 2004). When we modified the AP waveform to combine both the 15% decrease in amplitude and ~30% increase in width, we detected a reduced EPSC amplitude of approximately 60% (0.7 ± 0.2 nA, n = 4) of the control that was close to our experimental recordings (Fig. S3a, middle right). If we only increased the AP width and left the amplitude unchanged, the postsynaptic EPSC

dramatically increased to approximately 432% (4.6 ± 0.6 nA, n = 4) of control (Fig. S3a, right). To quantitatively describe how the AP waveform influences the postsynaptic response, we varied the AP amplitude (from 110 mV to 130 mV with a step of 5 mV) and half-width (from 0.48 to 0.88 ms with a step of 0.1 ms) and recorded the corresponding EPSC (Fig. S3b, c). The normalized EPSC showed a highly non-linear relationship versus the AP amplitude, with a power of 11.4 (EPSC = 1.06 * $AP_{amp}^{11.4}$, r^2 = 0.98, Fig. S3b, right), which is similar to a previous report(Wu et al., 2004). The relationship between the normalized EPSC amplitude and AP half-width also fit the linear regression with a slope of 4.1 in a double logarithmic scale (EPSC = 0.99 * $AP_{width}^{4.1}$, r^2 = 0.99, Fig. S3c, right). The quantitative relationship between EPSC and AP amplitude and width can help us understand the physiological effect of many related clinical anesthetics.

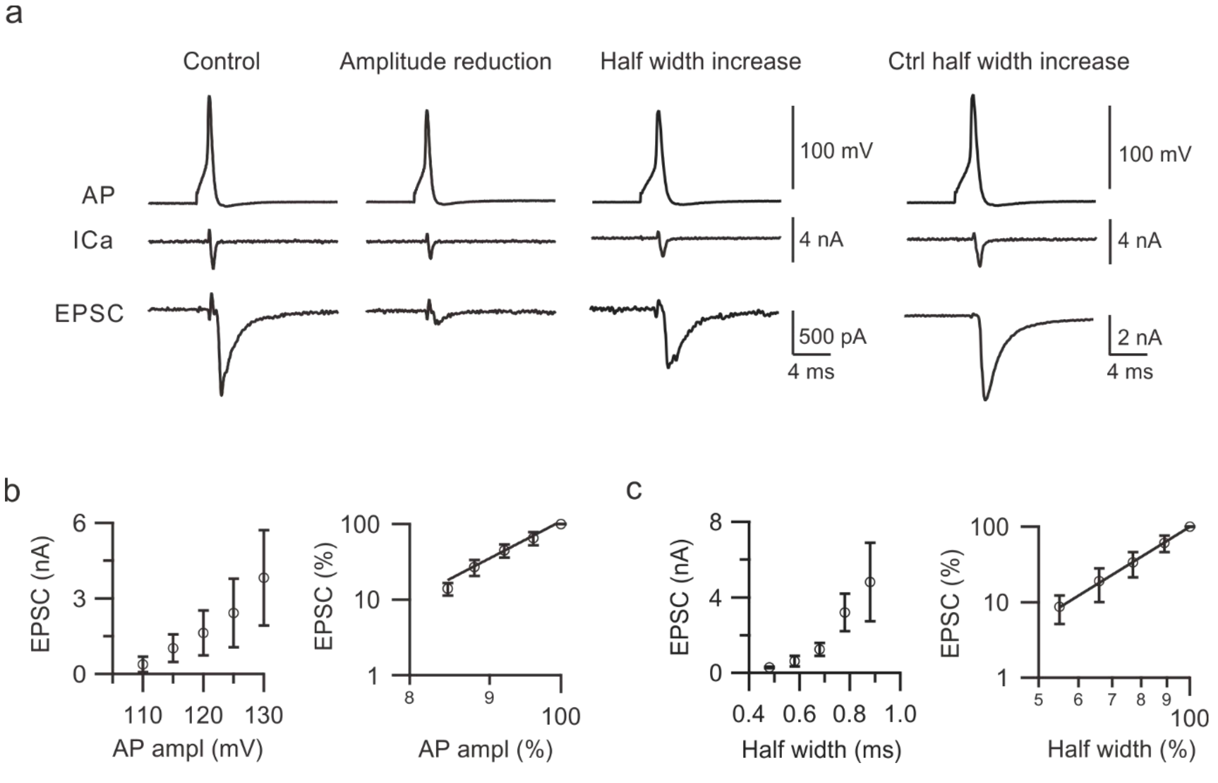

Figure S3. Relationship between the EPSC and presynaptic action potential waveform

a) Left: Sampled generated AP and its corresponding calcium current (ICa) and EPSC. Middle left: Similar to left, but the AP amplitude was reduced by 15%. Middle right:

Similar to middle left, but the AP half-width was increased by 30%. Right: Similar to left, but the AP half-width was increased by 30%.

b) Left: Relationship between the EPSC amplitude and presynaptic AP amplitude ranging from 110 to 130 mV with a step of 5 mV (n = 4). Right: Same data as in left but normalized to EPSC induced by the 130 mV AP amplitude.

c) Left: Relationship between the EPSC amplitude and presynaptic AP half-width ranging from 0.48 to 0.88 ms with a step of 0.1 ms (n = 4). Right: Same data as in left, but normalized to the EPSC induced by the 0.88 ms AP half-width.

V. Increased extracellular calcium can counterbalance propofol-inhibited exo-endocytosis and the EPSC

To address whether propofol can directly modulate vesicle exo-endocytosis, we increased the extracellular calcium to 3 mM to counterbalance the propofol-inhibited calcium influx. After $depol_{20ms}$, the calcium current in propofol-treated calyces was increased to a similar level as controls (2.0 ± 0.1 nA, n = 5; p = 0.8; Fig. 2E, F). The amount of exocytosis and $Rate_{endo}$ recovered to normal (exocytosis: 457 ± 20 fF, p = 0.5; $Rate_{endo}$: 37 ± 9 fF/s, p = 0.4; n = 5; Fig. 2E, F). The calcium influx, exocytosis, and endocytosis after $depol_{20msx10}$ were also similar to controls (calcium influx: 335 ± 32 pC, p = 0.8; exocytosis: 1517 ± 74 fF, p = 0.8; $Rate_{endo}$: 204 ± 17 fF/s, p = 0.2; n = 5; Fig. 2G, H), suggesting a calcium-dependent mechanism in propofol-inhibited vesicle exo-endocytosis. In addition, when the extracellular calcium was increased to 3 mM in propofol-treated calyces, the propofol-inhibited EPSCs also increased to a level similar to controls (control: 8.0 ± 1.0 nA, n = 5; propofol-treated: 6.6 ± 1.0 nA, n = 5, $p < 0.05$; high calcium: 7.6 ± 1.1 nA, n = 5, p = 0.4; Fig. S4). Taken together, these results suggest a predominate calcium-dependent presynaptic mechanism in propofol-inhibited synaptic transmission at the calyx of Held.

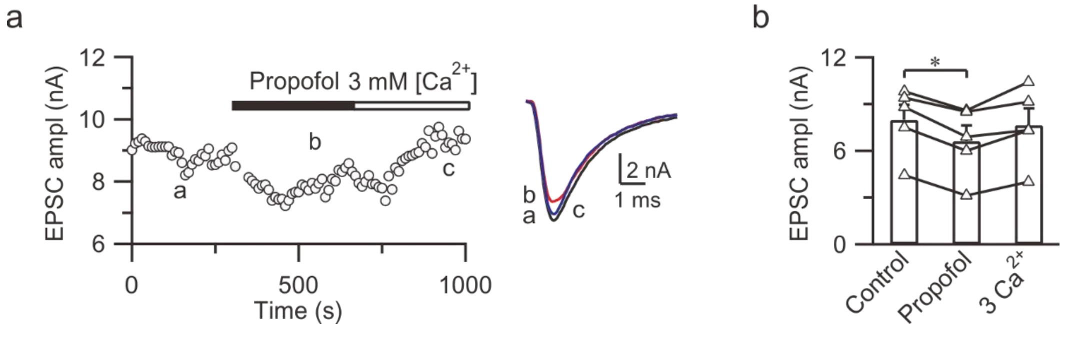

Figure S4. Increased extracellular calcium can counterbalance propofol-inhibited EPSC

a) Left: Sampled EPSC in controls, in the presence of 250 µM propofol, or in the presence of 250 µM propofol with 3 mM extracellular calcium calyces. Right: Sampled EPSCs from time point a (black), b (red), and c (blue) were superimposed.

b) Statistics for EPSC amplitude in controls (n = 5), in the presence of 250 µM propofol (n = 5), or in the presence of 250 µM propofol with 3 mM extracellular calcium (n = 5) calyces. * $p < 0.05$.

Reference List

Ishikawa, T., Nakamura, Y., Saitoh, N., Li, W.B., Iwasaki, S., and Takahashi, T. (2003). Distinct roles of Kv1 and Kv3 potassium channels at the calyx of Held presynaptic terminal. J Neurosci 23, 10445-10453.

Nakamura, Y., and Takahashi, T. (2007). Developmental changes in potassium currents at the rat calyx of Held presynaptic terminal. J Physiol 581, 1101-1112.

Wu, X.S., Sun, J.Y., Evers, A.S., Crowder, M., and Wu, L.G. (2004). Isoflurane inhibits transmitter release and the presynaptic action potential. Anesthesiology 100, 663-670.

Xue, L., and Wu, L.G. (2010). Post-tetanic potentiation is caused by two signalling mechanisms affecting quantal size and quantal content. J Physiol 588, 4987-4994.
